# Understanding the dimensions of a strong-professional identity: a study of faculty developers in medical education

**DOI:** 10.1080/10872981.2020.1808369

**Published:** 2020-08-14

**Authors:** Rosa Nelly Cavazos Montemayorr, Jose A Elizondo-Leal, Yoel Adbel Ramírez Flores, Ximena Cors Cepeda, Mildred Lopez

**Affiliations:** Tecnologico de Monterrey, Escuela de Medicina y Ciencias de la Salud, Monterrey, Mexico

**Keywords:** Medical educator, professional identity, faculty developers, strong-professional identity, higher education, educational innovation, educator identity

## Abstract

**Background:**

Faculty developers are regularly involved in training medical educators to enhance their teaching excellence through workshops and other formats. By exemplifying professional and institutional values, faculty developers may profoundly impact how other educators perceive their own professional identity.

**Objective:**

The objective of this study was to understand how the professional identity of faculty developers is formed.

**Design:**

A qualitative approach was used, with a semi-structured interview. The sample consisted of 10 medical educators. A deductive thematic analysis based on Bolivar et al. (2004a) model of professional identity formation for medical educators was carried out.

**Results:**

Self-image was impacted favorably through social recognition from students and peers, and the belief of having demonstrated professional competence through job assignments and enrollment in different leadership positions. The social relations to the center or department in which the faculty developer participates were strongly related to job satisfaction. Expectations about the future of the profession included positive attitudes toward change brought by generational differences. Regarding the process of construction of professional identity, life stories and dissimilar professional careers converge in the same educational setting. Faculty developers regularly resort to self-reflection, with a desire to continue learning and developing. They are resilient and purposeful, even in negative experiences that they have faced as identity crises. They share an awareness in building a legacy for the patients, their families, and the community through nurturing new generations of health-care practitioners.

**Conclusions:**

The interviewed faculty developers have a strong-professional identity that is characterized by a stable sense of self, strong behavioral repertoire, and key associations with a community of practice.

## Introduction

Professional identity is a concept that has been widely discussed for decades and is still vaguely understood. This identity evolves with time and work-related experiences [1]. It negotiates personal, ego, and social elements within one individual that participates in a professional setting [2]. A strong identity has been linked to job satisfaction determined by career planning and achieving intended goals [3].

For the medical educator, professional identity is the process of learning to teach and act as a teacher. Faculty developers, a special group of medical educators, are regularly involved in training medical educators to enhance their teaching excellence through workshops and other formats [4]. They partake in academic activities in which their commitment to the community is evident. They come from different backgrounds and personal experiences, as medical education is a field, not a discipline [5]. The experiences and choices they made to become faculty developers are inexorably linked to their identity and life story [6].

**Figure 1.**
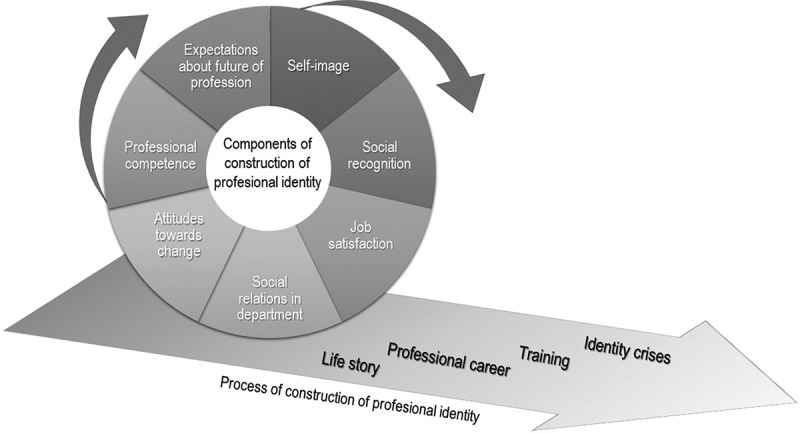
Dimensions of the professional identity of medical educator’s model.

Those components converge in a professional identity that is carried through three domains: role transitions, socialization, and identity work [7]. *Role transitions* prove that identities change as an individual makes progress in their career. Several authors describe also the *socialization* of the profession as the means in which organizations shape their member’s identity [8]. The *identity work* refers to how individuals actively construct their identity through an incremental process [9]. Reconciling these three domains, professional identity is described as composed by elements of self-image, social recognition, job satisfaction, social relations, attitudes toward change, professional competences, and expectations about future of the profession, illustrated in the wheel model from Figure 1. Individual constructions are unique and related to personal factors such as life story, professional career, training, and identity crises.

Faculty developers, typically in leadership roles in their institutions, have the responsibility to train the future generation of physicians, and their identity construction process is vital for medicine [10]. By exemplifying professional and institutional values, faculty developers may profoundly impact how other educators perceive their own professional identity [11]. The commitment that they have towards their learning community is tangible, which makes them in an interest group to study professional identity construction.

The objective of this study was to understand how the professional identity of faculty developers is formed. Two research questions were developed: what elements integrate the professional identity of faculty developers in medical education; and what is the process in which they construct their professional identity. At the first level, these professionals are in charge of helping other medical educators develop their professional identity. On a second level, doing so will enable individual faculty developers to better understand and improve their practice, as well as to optimize a formal training of competent faculty developers [12].

## Method

A qualitative approach is used to explore the interpretation that those in faculty development have of their professional identity. This type of qualitative study seeks to understand on a personal level the motives and beliefs that are behind the actions of the participants, through the collection of words and behaviors [13]. Data were collected through a semi-structured interview [14].

The sample technique used to recruit participants was convenience sampling, the participants belong to a medical school in which the research team was able to recruit participants through contact information available to members in the university. It consisted of 10 faculty developers with an average of 14.5 years of teaching experience. Inclusion criteria included a background on medicine, faculty development, and holding a leadership role in the university. Their roles were department head, basic science lecturer, and clinical teacher, to name a few. Table 1 characterizes these participants.

**Table 1.** Participants in the study.

Participant	Role	Gender
1	Department head	Female
2	Clinical teacher	Male
3	Department head	Male
4	Basic science lecturer	Female
5	Clinical teacher	Female
6	Clinical teacher	Female
7	Department head	Male
8	Basic science lecturer	Female
9	Basic science lecturer	Female
10	Department head	Female

The participants had postgraduate studies in the following proportions: 41.6% had a medical residency, 58.3% a master’s degree, and 33.3% a doctorate. The interviews were conducted in Spanish by two members of the research team and had a mean duration of 25 minutes. These researchers had previous experience in conducting structured interviews and have a background in psychology and education. They had performed a previous study on describing the maturity of the construction process of the professional identity of medical educators [15]. The interviewers did not know the participants directly but belong to the same university. Participants completed informed consent and had the opportunity to review the stated content and clarify the arguments presented, and if necessary amend or ask the interviewer to provide details.

The instrument and methodology used for the study are an adaptation of Steinert [4], which explores through open-ended questions the process of construction of the identity of these medical educators. As suggested by the author, several probes were used to boost follow-up questions based on examples that could illustrate their affirmations.

Interviews were audio-recorded and transcribed verbatim to organize and systematize the data for further analysis and interpretation [16]. The analysis strategy was deductive thematic analysis as five researchers read each of the transcripts, discussed their coding, and came to consensus of the coding of all transcripts [17]. Emergent themes were added to the codification scheme and further integrated into the resulting model.

The study was conducted in accordance with the Declaration of Helsinki, and the protocol was approved by the Ethics and Research Committee of Tecnologico de Monterrey.

## Results

This section presents the findings about faculty developers’ components of professional identity and their process of construction. Extracts from the different participants are used to exemplify each element and present a definition of the components. Those components intersect in a professional identity that is carried through different roads and pathways in an incremental process of construction, in a snowball effect.

### Components of construction of professional identity

#### Self-image

Self-image is the way the professionals perceive themselves based on social, emotional, and cognitive dimensions. It is a projection from the past and into the future [18]. Cote describes identity formation as a process that starts with professionals acquiring an assigned status; evolving later to an achieved professional with a position based on merits; and finally becoming a managed professional that reflects and strategically gains the approval of the community of practice [19]. Faculty developers describe a self-image made of external validation of society and students [9]. The participants mentioned that their roles as medical educators were earned based on merit and effort, and the community validated it [19]:

… with my little girl I am ‘the mother’, with my husband’s friends, I am ‘the doctor’s wife’. Here [in the school of medicine] it’s me, it’s only me. The positive, negative reviews that I have are something that I worked on, that I earned, that I cultivated (participant 4).

The result leads to an integrated version of reality, which is dynamic and evolves accordingly to the stage of their professional life. Even the participants that did not come from a health-related background have achieved the managed professional stage [19], where an individual is part of a community of ‘strangers’ because of gained approval [19]:

When I started there was a lot of rejection … it took me a semester until I really started working with them (participant 1).

#### Social recognition

Social recognition is multifactorial and is developed with time and experience. It is largely influenced by the differentiated values that the practitioners set in their working environment, both with patients and with fellow colleagues [11]. Participants described that this emerges from the interaction with students. When the students engaged with their experiences, it had a positive impact on the perception of their career:

We have many years of experience where many things have happened …, students appreciate [those stories] that they can listen from us (participant 5).

#### Job satisfaction

Job satisfaction is directly related to the desire to continue or leave their profession [20]. When a professional becomes dissatisfied the repercussions can be seen beyond the students’ experience, compromising even patient care [21]. Although most incentives are money or awards related, participants did not mention salary or monetary incentives guiding their satisfaction. Another important finding was that even when participants mentioned they were enrolled in many activities they did not refer to it as a burden. Participants emphasized that they enjoy being educators, even considering continue doing it in the long run:

The truth is that I like it … I think that I see myself doing this for longer (participant 8).

Another meaningful way was the establishment of social relationships with the interaction with people eager to learn.

[Teaching] gives a lot of meaning to my life, it keeps me alive … I feel that although we are very different, there is something that we share (participant 6).

#### Social relations in department

Social relations in the department refer to the place where the medical educator is active that feeling of belonging to a social group that shares characteristics and competences [18]. Friction can emerge from the extrinsic, social expectations of how teachers should act, and the intrinsic way in how they define who they are [22]. The participants described the responsibility of their role in the social structure, this hierarchy could favor teamwork when used to empower and contribute to the development of other educators:

Being in a leadership position in the department I come in contact with many professors … I am there to support them (participant 3).

This responsibility could be inherited or acquired, and participant dimension their contribution towards achieving the goals of the organization:

Recently, with all the changes in the educational program, everything became like a ‘Jenga game’ … I had to reimagine what was the best [curriculum] design … it was, it is, a very big responsibility (participant 10).

#### Attitude towards change

Attitudes toward change describe how professionals respond to transformations, both in their experiences with students and faculty and the innovations in their field [23]. Participants described proactive attitudes and positive feelings. These faculty developers had the will to make changes and practice continuous improvement.

As educators, there are a lot of things that you need to develop continuously (participant 6).

Participants showed awareness that teaching in the XXI century involves knowing the student, their habits, and their psychology:

Millennial students, generation z or whatever … have other interests. That does not mean that they study less, but they do have another way to learn. So, it is a challenge for us teachers. We are always trying to be in workshops and in training, not being afraid of the technological changes because it is the way to keep providing the student of the new generations (participant 8).

#### Professional competence

Professional competences encompass a variety of skills and abilities to work and make their teaching more effective, both for the students and educators. For example, conducting mentoring increases the skills of the professional to delve in empathy and understanding of the needs of the student experiences [24]. Participants described the knowledge and skills that are part of having a role in faculty development [11]. They described examples of how this acquisition process is not always conscious:

Sometimes it happens as time passes and as you get experience. Unintentionally it is happening (participant 2).

Other participants mentioned that being a physician does not prepare a professional to be an effective trainer:

I have heard my students talk about other professors, saying that they are very good [clinicians] … but their classes are terrible. So I think it has a lot to do with the way you communicate things. Being competent is not only knowing, it is crucial to know how to deliver it (participant 9).

#### Expectations about future of profession

Expectations about the future of profession are the goals and objectives that they hope to achieve following a certain period of time [95]. The future is something to look forward to, both regarding the personal life, future of medical students, and evolution of health sciences [11]. Faculty developers described professional models that govern and define current and future goals [9]. The participants emphasized the legacy and transcendence of their work:

You get to see how people evolve. You sow that seed, seeing that seed bloom helps you grow … it is very good feedback (participant 9).

Other participants described the long-run effect that their contributions on medical education have, even though they might not see the result at the end:

[Medical education] has great impact and transcendence because our students will impact many more individuals when they practice what they learned, towards their patients, their families, their clinics, office, operating room, whatever they do. Deep down, you know that you influenced that (participant 7).

### Process of construction of professional identity

#### Life story

Life story is composed of biographical life experiences that result in perspectives and intention to become, or not, a medical educator [11]. Life experiences influence the way they perceive themselves as humans and mentors [4]. Most of the interviews provided evidence of a career seeking compatibility with family life. One participant described changes in residency that impacted job opportunities:

I would say it was more about destiny. I got married and came to study here. I am from another city … as soon as I arrived, I was working. I taught Anatomy at a private university (participant 4).

Another important element was family as positive influence of perception of teaching as a profession:

I have always enjoyed teaching; I think it was because both of my parents were educators. I grew in a house where it was natural to seek transcendence in young generations through teaching (participant 1).

#### Professional career

Professional career begins with a stage of exploration in which the professionals may be enthusiastic about their profession, or clash when they find out that it does not match their ideals [25]. Eventually, professionals could reach a breakpoint where they tackle an identity crisis and reappraise their participation as educators, even triggering professional detachment. Interviewees presented this component as a narrative, reflecting stages, milestones, and changes of functions or job positions. Participants heavily relate the acquisition of their role in medical education to the time when they finished postgraduate studies. They had a progression of responsibilities as they acquired more experience:

Right now I am the director of postgraduate studies … I also participated as an educator in the gynecology and obstetrics program, then director of clinical sciences, until [I got] my current responsibilities (participant 3).

The participants refer to their time as students and reflect on their experiences.

My decision to become a medical educator was after completing two medical specialties and the Ph.D. I deliberately sought to join this university because it is where I did my undergraduate studies. I really enjoyed those sessions (participant 3).

#### Training

Training can be formal or informal. For most faculty developer experience is gathered over the years, as they build their courses, or through participation in faculty workshops [4]. Participants mentioned the importance of their development through formal and informal training experiences. They said that it was no longer required to be a physician to be enrolled in the faculty development of educators and that other professionals could contribute to the field, especially disciplines like pedagogy.

You can be a medical educator coming from the field of medicine, like a surgeon, nurse, dentist, psychologist, entering medical education or vice versa, that you are a teacher with a background in education and migrate to medical education (participant 9).

Some of the mentioned informal experiences that contribute to training were educational conferences and short-term faculty development workshops where you engage with other educators:

Educational innovation conferences are where you share … a lot of the innovation [practices] and this helps to focus on things that you may even thought about (participant 6).

#### Identity crises

Identity crises are important moments or milestones, usually associated with negative experiences that influence the professional to feel less qualified or even have less interest in their role [9]. They may begin as complaints about students, colleagues, or educational system [25] and eventually scale into a change of goals or even profession. Participants mentioned this theme as moments where they questioned their status, perceived a low self-image, and job satisfaction was low. Only two participants considered not teaching anymore. These participants were the youngest of the sample:

They may be paying [lots of money] but they don’t have the right to treat the teacher like that. For me, it was like: No, I don’t want to go back to teaching! There or anywhere. It was the truth, at that moment (participant 4).

The opposite was found in the majority of the participants; they even describe moments in which they embrace their role more:

I have been in teaching, in an administrative position and with my patients, but I had not realized how closely interrelated they are. I can no longer say that education is in a second place [of importance], it is at the same level that my clinical practice (participant 5).

Table 2 presents a synthesis of the themes that were found in the participant’s interview. The themes that were most represented by the data were: self-image, social relations in the department, attitude towards change, and professional competence, which were mentioned in every interview. The themes that were least represented in the interviews were: life story (40%), and identity crises (30%).

**Table 2.** Representation of the themes in the interviews.

	Dimensions	Representation inthe interviews
Components of construction of professional identity	Self-image	10 (100%)
	Social recognition	8 (80%)
	Job satisfaction	8 (80%)
	Social relations in department	10 (100%)
	Attitude towards change	10 (100%)
	Professional competence	10 (100%)
	Expectations about future of profession	5 (50%)
Process of construction of professional identity	Life story	4 (40%)
	Professional career	5 (50%)
	Training	6 (60%)
	Identity crises	3 (30%)

## Discussion

This study found that the professional identity of faculty developers is in constant construction, allowing them to identify and face new challenges with passion and enthusiasm. The approach in the study allowed the research team to collect feelings, emotions, and experiences associated with the role of faculty development and their unique way of appropriating of a professional identity.

Highly engaged individuals, such as faculty developers, have a strong professional identity that is accompanied by a stable sense of self, strong behavioral repertoire, and key associations with social and community networks.

Self-image was impacted favorably through social recognition from students and peers. Similarly, to what other authors found that belief grew through enrollment in different leadership roles and positions [26]. The social relations in their department influenced what the growth of the professional and development as an educator [27]. Contrary to what other authors have found, economical or monetary incentives were not important for them [9,19]. The themes of expectations about the future of profession included positive attitudes toward change and transcendence through the impact of new generations.

Regarding the process of construction of professional identity, life stories and dissimilar professional pathways and background converge in this same educational setting. As found in other studies, faculty developers regularly resort to self-reflection and looking at things critically, in the permanent desire to continue learning and developing [4]. They are resilient and purposeful, even in the negative experiences that they might have faced as crises. They share the awareness in providing and leaving a legacy to the patients, their families, and the community through nurturing new generations of health-care practitioners.

The conceptual understanding of the medical educator is evolving alongside the changes in the health-care system, education system, and society [12]. It has shifted to be more than just the development of knowledge and skills of clinical education, to a professional that brings to the role self-knowledge, deep understanding of self, and relationship with others [27]. The role of faculty developers has transformed as well, as they need to assist medical educators in bringing them to a conscious level and provide a safe environment for reflection [26].

This study has a number of limitations. The data were collected from a small number of faculty developers from one institution and in one country, which may not be generalizable to other faculty developers. However, it is consistent with the approaches of qualitative research to explore a phenomenon in depth. The participants in the sample were not necessarily diverse in terms of race or ethnicity. The length of the interviews was limited; therefore, it was hard exploring seven components and four processes with participants. The model is presented not as a generalized finding applicable for other programs or countries but as a model that can be assessed and adapted to particular contexts. Future studies could deepen on identifying facilitators and barriers which impacted career decisions of medical educators, analyze how belonging to a minority group could shape their identity, and the influence of established career paths on medical education. Identifying these elements is important to foster initiatives, programs, and reflection that would guarantee the future of medical education, through developing and attracting similar professionals to academic careers.
